# miR-140-5p regulates adipocyte differentiation by targeting transforming growth factor-β signaling

**DOI:** 10.1038/srep18118

**Published:** 2015-12-11

**Authors:** Xin Zhang, Ailing Chang, Yongmei Li, Yifei Gao, Haixiao Wang, Zhongshu Ma, Xiaoxia Li, Baoli Wang

**Affiliations:** 1 Collaborative Innovation Center of Tianjin Metabolic Diseases Hospital, Key Laboratory of Hormones and Development (Ministry of Health), Metabolic Diseases Hospital & Institute of Endocrinology, Tianjin Medical University, Tianjin 300070, China; 2 Division of Endocrinology, Tianjin Medical University General Hospital, Tianjin 300052, China; 3 School of Integrative Medicine, Tianjin Traditional Medical University, Tianjin 300193, China; 4 College of Basic Medical Sciences, Tianjin Medical University, Tianjin 300070, China

## Abstract

Recent emerging studies of miRNAs in adipocyte commitment provide new insights to understand the molecular basis of adipogenesis. The current study indicated that miR-140-5p was altered in primary cultured marrow stromal cells and established progenitor lines after adipogenic and/or osteogenic treatment. miR-140-5p was increased in adipose tissue in db/db obese mice *vs.* lean mice. Supplementing miR-140-5p activity induced stromal cell ST2 and preadipocyte 3T3-L1 to differentiate into mature adipocytes. Conversely, inhibition of the endogenous miR-140-5p repressed ST2 and 3T3-L1 to fully differentiate. By contrast, knockdown of the endogenous miR-140-5p enhanced osteoblast differentiation. Transforming growth factor-β receptor I (Tgfbr1) was shown to be a direct target of miR-140-5p. Supplementing miR-140-5p in ST2 reduced the level of TGFBR1 protein, while suppression of endogenous miR-140-5p increased TGFBR1. Overexpression of Tgfbr1 inhibited, whereas knockdown of Tgfbr1 promoted adipogenic differentiation of ST2 cells. Further investigation of mechanisms that control miR-140-5p expression revealed that C/EBPα induced transcriptional activity of the miR-140-5p promoter. Removal of the putative response element of C/EBP from the promoter abolished the enhancement of the promoter activity by C/EBPα, suggesting that C/EBPα transcriptionally controls miR-140-5p expression. Taken together, our study provides evidences that miR-140-5p regulates adipocyte differentiation through a C/EBP/miR-140-5p/TGFBR1 regulatory feedback loop.

It is believed that adipocyte and osteoblast originate from a common mesenchymal stem cell (MSC), thus a reciprocal and inverse relationship exists between adipogenesis and osteogenesis^1^. Commitment of marrow mesenchymal stem cells to adipocytes is governed by the coordination of peroxisome proliferator-activated receptor γ (PPARγ) and members of the CCAAT/enhancer binding protein (C/EBP) family^2^,^3^,^4^,^5^. *In vivo* and *in vitro* studies have shown that PPARγ is critical both for adipocyte differentiation and for major functions of mature adipocytes, including lipid metabolism, adipokine secretion, and insulin sensitivity^6^,^7^. Three members of C/EBP family, i.e., C/EBPα, C/EBPβ, and C/EBPδ, have been found to work together with PPARγ to fully exert their adipogenic roles^8^,^9^,^10^,^11^,^12^. Osteogenesis is also a highly coordinated process and is governed by the activation of Wnt/β-catenin signaling and the expression of several master transcription factors including Runt-related transcription factor 2 (Runx2), osterix (Osx) and distal-less homeobox 5 (Dlx5)^13^,^14^.

Recent emerging studies have shown that miRNAs are able to indirectly regulate adipogenic and/or osteoblast differentiation of MSCs by targeting various genes that may be involved in balancing self-renewal and stem cell differentiation. miRNA family 27 (miR-27a and miR-27b) has been found to be able to negatively regulate adipogenic differentiation of 3T3-L1 preadipocytes^15^,^16^. miR-155, miR-221 and miR-222, have also been reported to be the negative regulators of the adipogenic programming of human bone-marrow-derived stromal cells. By contrast, ectopic expression of miR-637, miR-204 and miR-211 induced osteogenesis and at the same time blunted adipogenesis, thus may play a key role in balancing the commitment potential of MSCs. While miR-637 directly targeted osteogenic transcription factor Osterix^17^, miR-204 and its homolog miR-211 functioned via binding the 3′-UTR of another key modulator of osteoblast differentiation, runt-related transcription factor 2 (Runx2)^18^. Our group has recently demonstrated that miR-20a, miR-30e and miR-223 are novel players that contribute to adipogenesis^19^,^20^,^21^.

microRNA-140 (miR-140), generated from the *Mir140* gene, was previously believed to be abundantly and relatively specifically expressed in chondrocytes. The *in vivo* function of miR-140 was demonstrated by Miyaki’s group, who created miR-140 knockout and transgenic mice^22^. The miR-140 null mouse had a mild developmental phenotype in the skeleton, potentially via reduced growth plate chondrocyte proliferation, but displayed a premature osteoarthritis phenotype driven at least in part by an increase in expression of *Adamts5*, which was shown to be a direct target of miR-140. Conversely, the transgenic mouse overexpressing miR-140 in cartilage displayed no skeletal phenotype during development but was resistant to antigen-induced arthritis. Recently, miR-140 was identified as a direct downstream component of the BMP4 signaling pathway during the commitment of C3H10T1/2 cells to adipocyte lineage^23^. Overexpression of miR-140 in C3H10T1/2 cells promoted adipocyte differentiation, whereas knockdown of its expression led to impairment of adipogenesis. Further studies suggested that the anti-adipogenic factor osteopetrosis associated transmembrane protein 1 (Ostm1) is a bona fide target of miR-140^23^. The authors used a miR-140 precursor expression plasmid that overexpresses both miR-140-5p and miR-140-3p, making it unclear which one was exactly the contributor. At least, one could not exclude the possibility that miR-140-3p might also play a role in adipogenesis since miR-140-3p potentially targets several adipogenic or osteogenic factors like fibroblast growth factor 9 (FGF9), Wnt5 and TGF-β3. Moreover, little is known about the upstream mechanisms that control miR-140-5p expression during adipogenesis. In the present study, we provide novel evidences demonstrating that miR-140-5p is regulated by C/EBPα and is capable of controlling adipogenesis and osteogenesis via direct regulation of transforming growth factor β receptor 1 (Tgfbr1).

## Results

### miR-140-5p was upregulated during adipocyte formation

**Figure 1:**
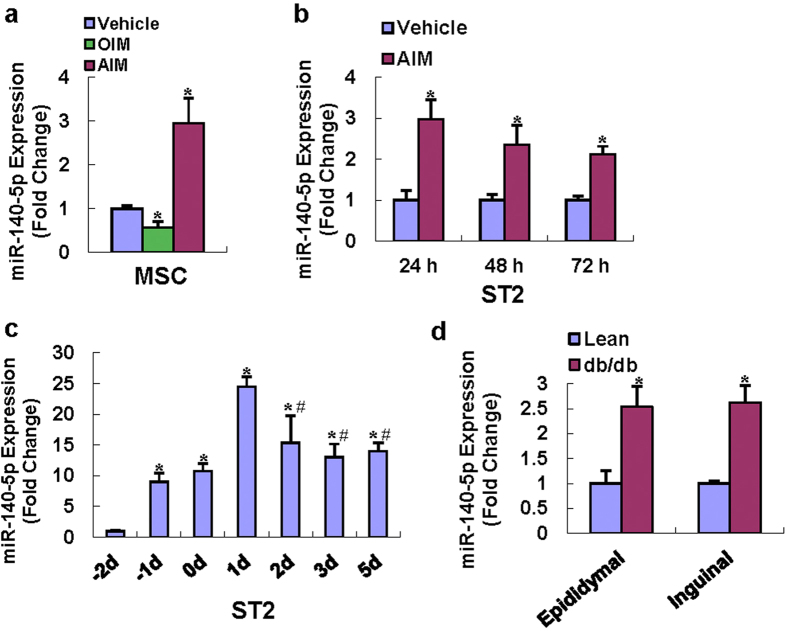
miR-140-5p was induced during adipocyte formation and reduced during osteoblast differentiation. qRT-PCR showed that adipogenic treatment (AIM) of primary marrow stromal cells for 72 h induced miR-140-5p, while osteogenic treatment (OIM) reduced miR-140-5p level (**a**). miR-140-5p was also induced in ST2 24-72 h after treatment with AIM (**b**). The level of miR-140-5p expression was also checked during a full differentiation time course (**c**). miR-140-5p expression was analyzed by qRT-PCR in db/db obese mice *vs.* genetically matched lean mice (n = 3 in each group) (**d**). Values represent the mean ± SD of three experiments. a, b: *Significant *vs.* vehicle treatment, p < 0.05. c: *Significant *vs.* −2d, ^#^Significant *vs.* 1d, p < 0.05. d: *Significant *vs.* lean mice, p < 0.05.

We previously demonstrated in the microRNA array analysis that the miR-140-5p was increased in primary cultured marrow stromal cells after adipogenic treatment as compared with vehicle treatment^19^. In the current study, we validated the expression profiles of miR-140-5p using qRT-PCR and showed that miR-140-5p was induced by 3-fold 72 h after adipogenic treatment versus vehicle treatment, and reduced by 40% 72 h after osteogenic treatment (Fig. 1a). Moreover, the expression of miR-140-5p was also induced in ST2 cells after adipogenic treatment at all the indicated time points (Fig. 1b). We also checked the level of miR-140-5p expression in ST2 during a full differentiation time course. As illustrated in Fig. 1c, the level of miR-140-5p expression increased dramatically when the cells reached confluence. 1 day after induction, it reached peak and then decreased during terminal adipocyte differentiation. In order to gain insights into the potential biological relevance of miR-140-5p in the regulation of adipose tissue *in vivo*, we examined the expression of miR-140-5p in the genetically obese db/db mice. Compared with the genetically matched lean mice, the expression level of miR-140-5p was increased in both epididymal and inguinal adipose tissue of the db/db mice (Fig. 1d). These results suggested that miR-140-5p might have a regulatory role in adipocyte and/or osteoblast commitment.

### Supplementing miR-140-5p activity in progenitor cells promoted adipocyte differentiation

The effect of miR-140-5p on the differentiation of stromal ST2 cells was examined. We first examined if miR-140-5p alone could induce differentiation without adipogenic treatment. Supplementing miR-140-5p alone could not promote ST2 cells to differentiate 9 days after transfection (Supplementary Fig. S1a,b). qRT-PCR revealed that it slightly increased the expression of adipogenic factors including PPARγ, adipocyte protein 2 (aP2) and adipsin (Supplementary Fig. S1c).

**Figure 2:**
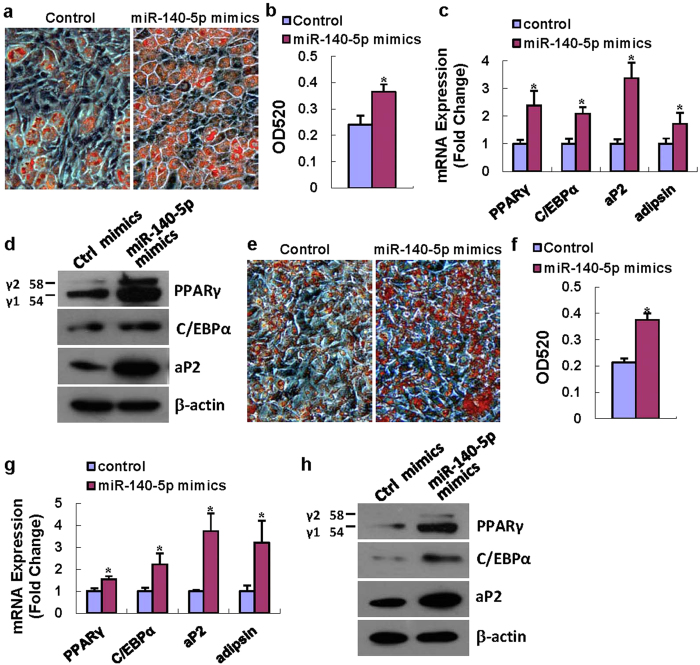
Supplementing miR-140-5p activity promoted adipocyte differentiation. miR-140-5p mimics transfection induced adipocyte formation from ST2 in the presence of AIM. Oil-Red O extracted with isopropanol was measured at OD520 (**a,b**). The mRNA levels of PPARγ, C/EBPα, aP2 and adipsin (**c**) and protein levels of PPARγ, C/EBPα and aP2 (**d**) were induced after transfection of miR-140-5p mimics. miR-140-5p mimics also induced adipocyte formation from 3T3-L1 cells (**e–h**). Values represent the mean ± SD of three experiments. *Significant *vs.* negative control transfection, p < 0.05.

However, when used in presence of adipogenic agents, miR-140-5p substantially induced adipocyte formation. As shown in Fig. 2, miR-140-5p mimics transfection into ST2 cells led to a significant increase of differentiated adipocyte numbers (52% increase in oil-red O staining compared to control transfection) (Fig. 2a,b). Accordingly, the mRNA levels of adipogenic transcription factors and marker genes, including PPARγ, C/EBPα, aP2 and adipsin, were increased by 2.4, 2.1, 3.4 and 1.7-fold *vs.* negative control, respectively, 48 h after adipogenic treatment (Fig. 2c). Consistently, the protein levels of PPARγ, C/EBPα and aP2 were increased in ST2 cells 72 h after transfection with miR-140-5p mimics as compared to control mimics transfection (Fig. 2d).

We further demonstrated that miR-140-5p mimics also significantly enhanced adipogenic differentiation from preadipocyte 3T3-L1 cells (75% increase in oil-red O staining compared to control transfection) (Fig. 2e,f). In 3T3-L1 cells, the mRNA levels of PPARγ, C/EBPα, aP2 and adipsin were increased by 1.6, 2.2, 3.8 and 3.2-fold following miR-140-5p transfection as compared to negative control transfection, respectively, 48 h after adipogenic treatment (Fig. 2g). Moreover, miR-140-5p mimics also induced the protein levels of PPARγ, C/EBPα and aP2 72 h after adipogenic treatment as compared to negative control transfection (Fig. 2h).

### Inhibition of endogenous miR-140a-5p reduced adipocyte differentiation

**Figure 3:**
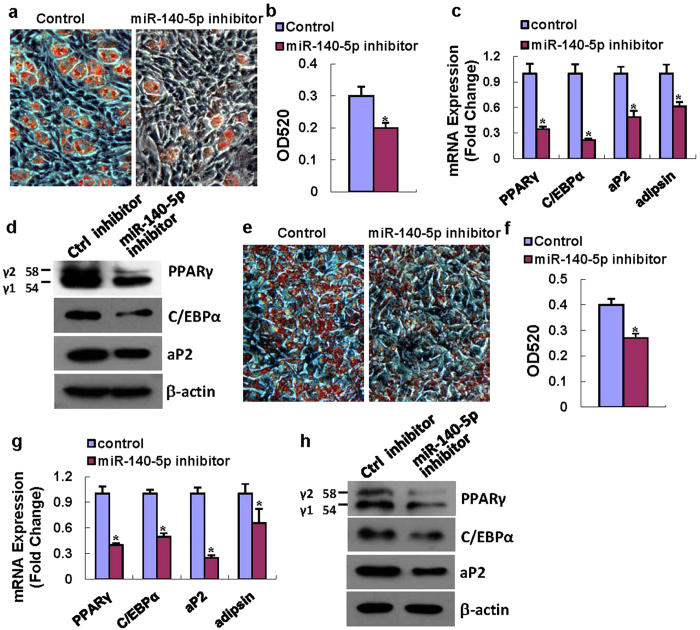
Inactivation of miR-140-5p reduced adipocyte differentiation. miR-140-5p inhibitor transfection blocked the adipocyte formation (**a,b**). Transfection of miR-140-5p inhibitor decreased the mRNA expression levels of PPARγ, C/EBPα, aP2 and adipsin at 48 h (**c**), and reduced protein levels of PPARγ, C/EBPα and aP2 at 72 h after adipogenic treatment (**d**). miR-140-5p inhibitor also inhibited adipocyte formation from 3T3-L1 cells (**e**–**h**). Values represent the mean ± SD of three experiments. *Significant *vs*. negative control transfection, p < 0.05.

Transfection of miR-140-5p inhibitor substantially blocked the differentiation of ST2 cells into adipocytes in presence of adipogenic treatment. Oil-red O quantification revealed a 35% decrease after miR-140-5p inhibitor transfection compared to control transfection (Fig. 3a,b). Consistently, the expression levels of the adipogenic factors were all downregulated by the miR-140-5p inhibitor compared to control transfection. Briefly, miR-140-5p inhibitor reduced the mRNA levels of PPARγ, C/EBPα, aP2 and adipsin by 65%, 78%, 51% and 39%, respectively, 48 h after adipogenic treatment (Fig. 3c). The protein levels of PPARγ, c/EBPα and aP2 were substantially decreased in cells transfected with miR-140-5p inhibitor 72 h after adipogenic treatment (Fig. 3d).

Furthermore, miR-140-5p inhibitor also blocked adipocyte formation from 3T3-L1 cells as compared to control transfection (Fig. 3e,f). Consistently, miR-140-5p inhibitor transfection significantly reduced the mRNA levels of adipogenic factors 48 h after adipogenic treatment, and decreased the protein levels of the adipogenic factors 72 h after adipogenic treatment in 3T3-L1 cells (Fig. 3g,h).

### Inhibition of endogenous miR-140a-5p promoted osteoblast differentiation

**Figure 4:**
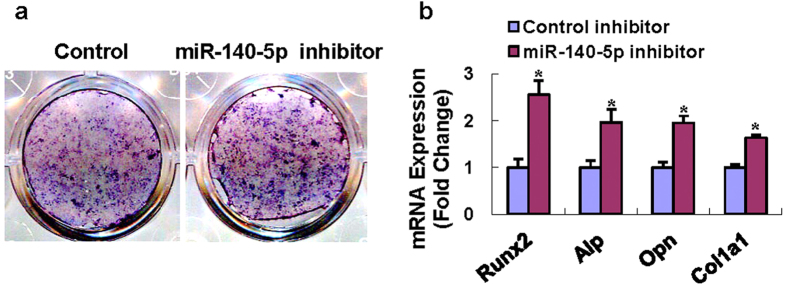
Inhibition of miR-140-5p in ST2 enhanced osteoblast differentiation. miR-140-5p inhibitor transfection enhanced ALP staining (**a**), increased mRNA levels of Runx2, Alp, osteopontin (Opn) and Col1a1 (**b**) in ST2 cells after osteogenic treatment. *Significant vs. negative control, p < 0.05.

To investigate if miR-140-5p plays a role in regulating osteoblast differentiation, we altered the level of miR-140-5p in stromal ST2 by transfecting the miR-140-5p inhibitor into the cells. Compared to the controls, miR-140-5p inhibitor enhanced alkaline phosphatase (ALP) staining 14 days after osteogenic treatment (Fig. 4a), suggesting the inhibitory role of miR-140-5p in osteoblast differentiation. Consistently, miR-140-5p knockdown significantly promoted the mRNA levels of Runx2, Alp, osteopontin and collagen type I (Col1a1) at day 14 (Fig. 4b).

### Tgfbr1 was a direct target of miR-140-5p

**Figure 5:**
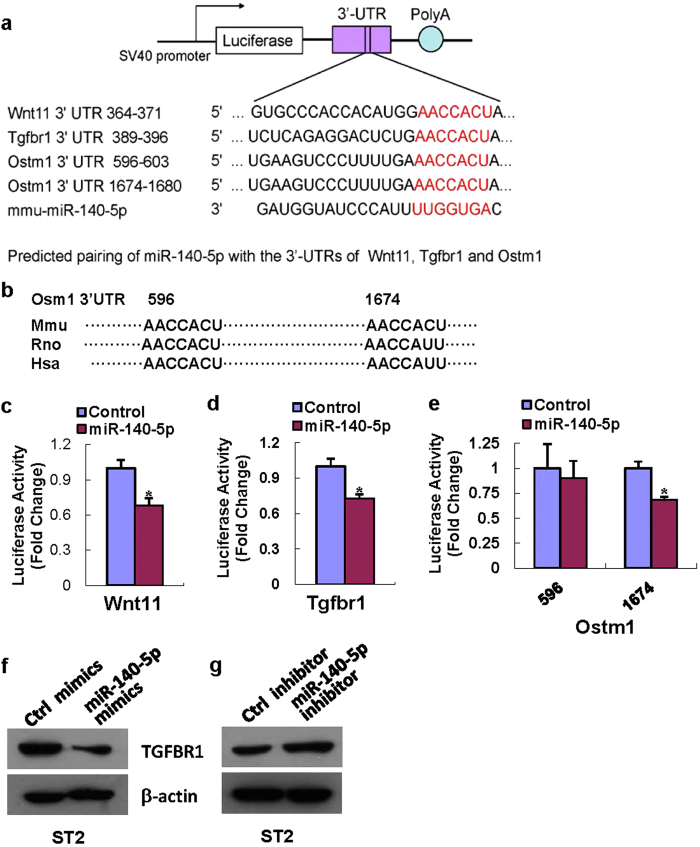
miR-140-5p directly targeted Tgfbr1. The 3′-UTR fragments of Wnt11, Tgfbr1 and Ostm1 were PCR-amplified and cloned (**a**), and the conservation of the two potential targeting sites on Ostm1 are shown (**b**). The increase of miR-140-5p in AD-293 decreased the luciferase activity of Wnt11 and Tgfbr1 3′-UTR constructs (**c,d**). It also decreased the luciferase activity of Ostm1 3′-UTR construct with site 1674–1680 but not that with 596–603 (**e**). miR-140-5p mimics transfection reduced, and miR-140-5p inhibitor transfection induced TGFBR1 protein level in ST2 cells (**f,g**). Values represent the mean ± SD of three experiments. *Significant vs. control transfection, p < 0.05.

It is well known that miRNAs function through blocking the translation of their target mRNAs. Several regulators of adipocyte and osteoblast differentiation including wingless-type MMTV integration site family member 11 (Wnt11), Tgfbr1 and Ostm1 were predicted to be the potential targets of miR-140-5p by using online programs. The reporter constructs of their 3′-UTRs were made and their potential pairings with miR-140-5p are shown in Fig. 5a. There are two potential targeting sites on 3′-UTR of Ostm1, with one conserved among mammals (position 596–603) and the other one not (position 1674–1680) (Fig. 5b). Luciferase assay showed that miR-140-5p mimics significantly reduced the luciferase activity of Wnt11 3′-UTR reporter (Fig. 5c) and Tgfbr1 3′-UTR reporter (Fig. 5d) in AD-293 cells compared to negative control. Liu *et al.* demonstrated that the site 1674–1680 of Ostm1 3′ UTR was the direct targeting site of miR-140, but they did not examine the site 596–603^23^. In our study, miR-140-5p mimics also decreased the luciferase activity of the Ostm1 3′-UTR construct with site 1674–1680 but not the construct with 596–603 (Fig. 5e), indicating that the conserved site is a pseudotarget.

These results suggested that Tgfbr1 is the direct target of miR-140-5p. Furthermore, supplementing miR-140-5p activity in ST2 cells using the synthetic mimics reduced the level of TGFBR1 protein (Fig. 5f). In contrast, suppression of endogenous miR-140-5p using the synthetic inhibitor resulted in an increase of TGFBR1 protein level as compared to control transfection (Fig. 5g).

### Perturbation of Tgfbr1 altered adipocyte differentiation

**Figure 6:**
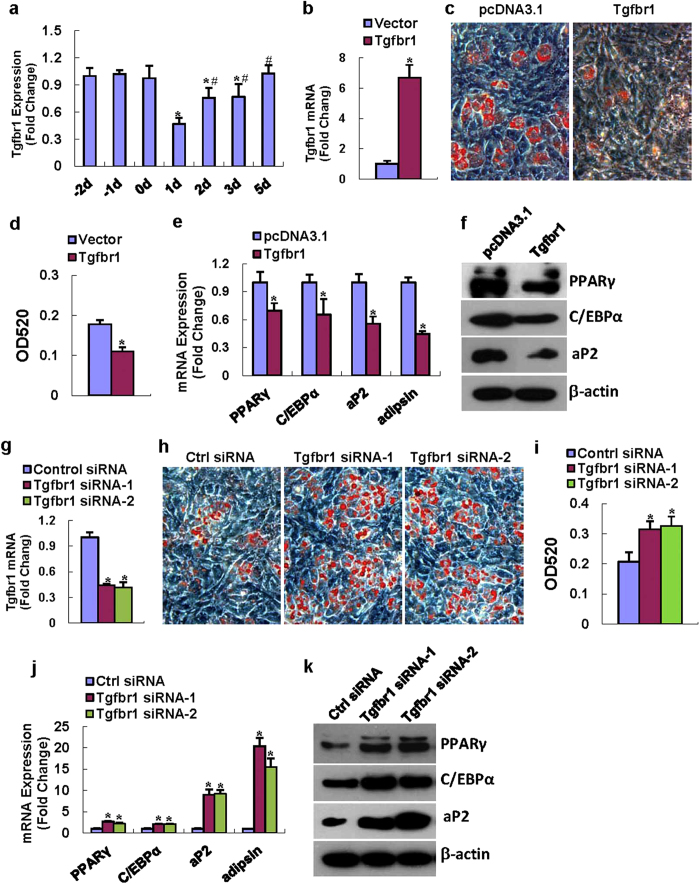
TGFBR1 negatively regulated adipogenesis. The mRNA expression pattern of Tgfbr1 was examined in ST2 during differentiation (**a**). The Tgfbr1 expression plasmid overexpresses Tgfbr1 mRNA in ST2 cells (**b**). Overexpression of Tgfbr1 in ST2 decreased adipocyte formation (**c,d**). The mRNA and protein levels of PPARγ, C/EBPα, aP2 and adipsin were inhibited (**e,h**). In contrast, transfection of the two Tgfbr1 siRNAs both efficiently knockdown mRNA level of Tgfbr1 (**g**), promoted adipocyte differentiation (**h,i**), and induced the mRNA and protein levels of PPARγ, C/EBPα, aP2 and adipsin in ST2 cells (**j,k**). Values represent the mean ± SD of three experiments. (**a**) *Significant *vs.* -2 day, p < 0.05, ^#^Significant *vs.* 1 day, p < 0.05; (**b–j**) *Significant *vs.* control transfection, p < 0.05.

It is well known that TGF-β signaling inhibits adipogenesis *in vitro* and *in vivo*^24^. However, it is unclear whether TGFBR1 directly regulates adipocyte formation from mesenchymal cells. We examined the expression pattern of Tgfbr1 during adipocyte formation from ST2 cells. As shown in Fig. 6a, tgfbr1 expression decreased 1 day after adipogenic induction and then increased from day 2 until day 5 when its level restored to normal. We then carried out Tgfbr1 gain-of-function and loss-of-function studies. The transfection of the Tgfbr1 expression plasmid in ST2 increased Tgfbr1 mRNA by 7-fold compared to empty vector transfection (Fig. 6b). In the presence of adipogenic medium, Tgfbr1 overexpression significantly inhibited adipocyte formation from ST2 cells (Fig. 6c,d). Consistently, the mRNA levels of PPARγ, C/EBPα, aP2 and adipsin were all decreased upon Tgfbr1 overexpression as compared to control transfection (Fig. 6e). The protein levels of PPARγ, C/EBPα and aP2 were also downregulated after Tgfbr1 overexpression (Fig. 6f). By contrast, the transfection of the two independent siRNAs targeting different regions of Tgfbr1 efficiently downregulated the endogenous mRNA level of Tgfbr1, suggesting they work well in the stromal cells (Fig. 6g). Furthermore, in the presence of adipogenic medium, the knockdown of the Tgfbr1 level by the two siRNAs dramatically enhanced formation of oil-red O positive adipocytes as compared to control siRNA transfection (Fig. 6h,i). The mRNA and protein levels of the adipogenic factors were substantially enhanced following Tgfbr1 knockdown by the two siRNAs (Fig. 6j,k).

### C/EBPs regulated miR-140-5p expression in mesenchymal progenitor cell

**Figure 7:**
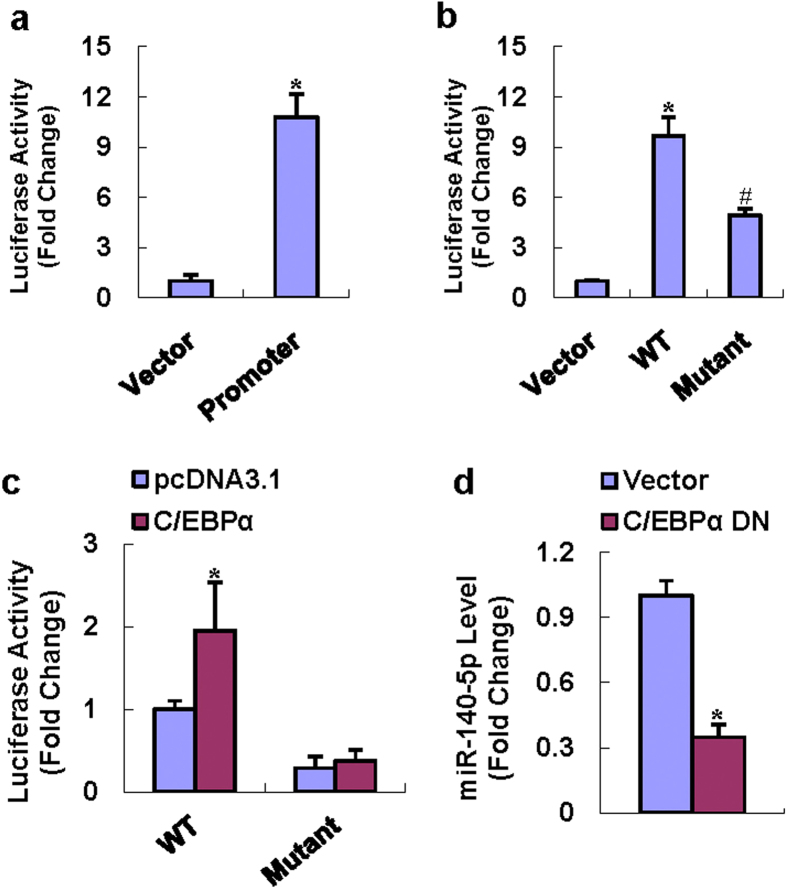
C/EBPs transactivated the miR-140-5p promoter. The 1.8-kb promoter fragment of miR-140-5p showed transcriptional activity in 3T3-L1 cells (**a**). Point mutation of the *miR140/Luc* construct at −281 nt decreased the promoter activity (**b**). Transfection of C/EBPα enhanced luciferase activity of the WT miR-140-5p promoter construct, but did not affect the mutant construct (**c**). The transfection of dominant-negative form of C/EBPα decreased the level of miR-140-5p (**d**). *Significant vs. empty vector, p < 0.05; ^#^Significant vs. WT promoter, p < 0.05.

To clarify the mechanisms that might regulate miR-140-5p expression, the promoter construct of miR-140-5p was made as described by Yang^25^. As shown in Fig. 7a, the promoter construct *miR140/Luc* had transcriptional activity in 3T3-L1 cells, showing 11-fold increase *vs.* the promoterless pGL3-basic. The prediction of transcription factor binding sites was performed with TFSEARCH (http://www.cbrc.jp/research/db/TFSEARCH.html) and TFBIND (http://tfbind.hgc.jp/), which revealed a putative C/EBP binding motif within the proximal promoter at −281 nt (CCCTTTCACCAGCC) (Fig. 7B), indicating that miR-140-5p might be regulated by C/EBPs. To assess the importance of the putative C/EBP binding site we made the point mutation within the *miR140/Luc* promoter reporter construct. The removal of the C/EBP binding site significantly reduced the transcriptional activity of the promoter (Fig. 7b). We further investigated whether C/EBPs activate the miR-140-5p promoter at the putative site. C/EBPα significantly induced the transcriptional activity of the WT promoter construct, while had no effect on the mutant promoter construct (Fig. 7c). Consistent to this, transfection of the dominant-negative construct of C/EBPα significantly decreased the expression level of miR-140-5p in 3T3-L1 cells 72 h after transfection (Fig. 7d).

## Discussion

In the present study, we found that the expression of miR-140-5p was increased either *in vitro* in stromal cells during adipogenesis, or *in vivo* in adipose tissue of db/db obese mice. Moreover, it was decreased in primary stromal cells during osteogensis, suggesting that miR-140-5p might play a role in adipogenesis and/or osteogenesis.

To delineate the precise role of miR-140-5p in cell fate decision, we tested the effects of miR-140-5p on adipocyte differentiation. Our data showed that supplementing miR-140-5p in undifferentiated adipogenic progenitors using synthetic miR-140-5p mimics potentiated the formation of adipocytes in presence of adipogenic treatment. Conversely, inactivating miR-140-5p in adipogenic progenitors using miR-140-5p inhibitor blocked differentiation of adipocytes. Concomitantly, knockdown of miR-140-5p using the inhibitor in stromal ST2 potentiated osteoblast differentiation. These findings provided evidences that miR-140-5p reciprocally regulates adipocyte and osteoblast differentiation from progenitor cells.

miRNAs finely modulate osteoblast/adipocyte differentiation through their direct targeting of diverse signaling molecules and pathways that include bone morphogenetic protein 2 (BMP2)/Smad, Wnt/β-catenin and Runx2 signaling pathways^26^,^27^,^28^,^29^. In an attempt to gain more insights into the regulatory mechanisms that control adipocyte differentiation by miR-140-5p, we analysed the potential targets using Targetscan, PicTar, and miRDB. Several important factors that might have contributions to the differentiation of osteoblast and/or adipocyte, i.e., Wnt11, Tgfbr1 and Ostm1, were predicted as the potential targets. Liu *et al.* demonstrated that the poorly conserved site 1674–1680 of Ostm1 3′ UTR was the direct targeting site of miR-140^23^. In our study, we also demonstrated miR-140-5p targeted this site but not the conserved site 596–603, indicating that the conserved site is a pseudotarget. The luciferase assay in our study also showed that miR-140-5p altered the luciferase activity of the 3′-UTR reporters of Wnt11 and Tgfbr1, suggesting they are the direct targets.

Wnt11 signals through both canonical and noncanonical pathways^30^ and is up-regulated during osteoblast differentiation and fracture healing^31^. Friedman *et al.* demonstrated that Wnt11 overexpression in preosteoblast increases β-catenin accumulation and promotes bone morphogenetic protein (BMP)-induced expression of alkaline phosphatase and mineralization^32^. Moreover, Wnt11 increases expression of R-spondin 2 (Rspo2), a secreted factor known to enhance Wnt signaling. The study concluded that Wnt11 signals to activate osteoblast differentiation through β-catenin, and Rspo2 expression^32^.

Of note, Wnt11 mRNA remains at a quite low expression level in the adipogenic cell lines we used before and after adipogenic treatment (data not shown). Thus it might not be critical during adipogenesis. By contrast, Tgfbr1 has much higher expression level in the adipogenic cell lines (data not shown). Furthermore, supplementing miR-142-5p in ST2 cells reduced, while silencing miR-142-5p induced the protein levels of TGFBR1. These data identified Tgfbr1 as a direct target of miR-140-5p.

TGF-β can provide competence for early stages of chondroblastic and osteoblastic differentiation, but it inhibits adipogenesis and late-stage osteogenesis^24^,^33^. TGF-β signaling begins with binding of TGF-β ligands to type II serine/threonine kinase receptor termed TGFBR2, which then phosphorylates and activates type I serine/threonine kinase receptor. The activated type I receptor activates Smad proteins that regulate transcription. Up to now, it is unclear whether TGFBR1 directly regulates adipocyte formation from mesenchymal cells. In the present study, we demonstrated TGFBR1 acts as a player in adipogenesis in murine progenitor cells. The knockdown of Tgfbr1 in the stromal ST2 cells enhanced the differentiation into mature adipocytes. By contrast, enforced expression of Tgfbr1 in ST2 blocked adipocyte differentiation. These data provide evidences that the cell fate decision of miR-140-5p may depend upon its inhibitory effect on TGF-β signaling, hence driving the progenitor cells to differentiate into adipocytes.

To gain further insight into the control of miR-140-5p expression and function, we cloned the promoter of miR-140-5p and examined cis-acting elements within the proximal promoter using TFSEARCH and TFBIND programs and found a potential binding site for C/EBPs within the proximal promoter. In our study, the WT promoter construct that harbored the C/EBP binding sequence showed transcriptional activity in 3T3-L1 cells, which was further stimulated following C/EBPα overexpression. Consistently, dominant negative form of C/EBPα downregulated the expression level of miR-140-5p. Moreover, point mutation of the C/EBP binding sequence at −281 nt was made and the mutant construct showed less transcriptional activity. Of more interest, the mutant failed to respond to C/EBPα transcription factor. We thus draw the conclusion that C/EBPs bind to the proximal promoter of miR-140-5p to positively regulate the expression of miR-140-5p.

The data we have shown in this paper tends to develop a model for miR-140-5p expression and function. Adipogenic signals lead to the activation of C/EBPs transcription factors in progenitor cells. In addition to working together with PPARγ to induce the coding genes essential for adipocyte differentiation, C/EBPs may also transactivate miR-140-5p promoter to increase the miR-140-5p level. In turn, miR-140-5p may further promote adipocyte differentiation and maintain the levels of C/EBPs mRNA and protein levels via repressing TGFBR1. Thus, we propose that a unique autoregulatory feedback loop exists among C/EBPs, miR-140-5p and TGFBR1. The current study also suggests that miR-140-5p is an attractive potential target for new therapies aimed at controlling metabolic disorders like osteoporosis and obesity.

## Methods

### Mice

10-week-old db/db mice and their genetically matched lean littermates were purchased from Model Animal Research Center of Nanjing University (Nanjing, China). RNA was extracted from epididymal and inguinal adipose tissue. qRT-PCR was done to examine the expression level of miR-140-5p. All the experiments involving animals were carried out in accordance with the Chinese guidelines for animal welfare and experimental protocol, and were approved by the Animal Care and Use Committee of the Metabolic Diseases Hospital, Tianjin Medical University.

### Cells

Bone marrow stromal cells were isolated from femurs and tibias of 4-week-old *C57* mice and cultured in α-MEM containing 10% FBS in 25 cm^2^ flasks as previously described^19^,^34^. For osteogenic differentiation, 80% confluent cells were treated with osteogenic medium (OIM, *α*-MEM containing 10% FBS, 50 μg/mL ascorbic acid and 5 mmol/L β-glycerophosphate) for 3 days followed by miRNA isolation. For adipogenic differentiation, confluent cells were cultured in adipogenic medium (AIM, *α*-MEM containing 10% FBS, 0.5 *μ*M dexamethasone, 0.25 mM methylisobutylxanthine, 5 *μ*g/ml insulin, and 50 *μ*M indomethacin) for 3 days followed by miRNA isolation.

3T3-L1 and ST2 cells were maintained in DMEM supplemented with 10% FBS. When the cells reached 100% confluence, AIM was added to induce adipocyte formation. When ST2 cells reached 80% confluence, OIM was added to induce osteoblast differentiation.

### Quantitative RT-PCR

We previously examined the miRNA expression profiles in primary cultured marrow stromal cells after adipogenic and osteogenic treatment^19^. In this study, we validated the expression levels of miR-140-5p by using a qRT-PCR system (Genecopoeia, Germantown, MD)^35^. Briefly, RNA was extracted using a miRNA isolation kit (Omega Bio-Tek, Norcross, GA, USA). 1 μg RNA was reverse-transcribed into cDNA using the RT primer. Subsequently, miRNAs were PCR-amplified on a real time fluorescent PCR cycler using specific forward primers and a universal reverse primer. The qRT-PCR consisted of 40 cycles (95 °C for 10 s, 60 °C for 10 s, and 72 °C for 10 s) after an initial denaturing step (95 °C for 2 min). The expression levels were normalized against U6, and measured by the comparative Ct (ΔΔCt) method^36^.

For mRNA expression analysis, total cellular RNA was isolated and reverse transcribed to cDNA. Real-time PCR amplifications were performed using SYBR green real-time PCR kit (Shenggong Biotech Company, Shanghai, China). The primers are listed in Supplementary Table 1. The expression levels of the target genes were normalized to that of β-actin.

### Transfections

50 nM mimics or 75 nM inhibitor of miR-140-5p (Genepharma, Shanghai, China) was transfected into cell cultures using lipofectamine RNAi-max (Invitrogen, Carlsbad, CA, USA) to activate or inactivate miR-140-5p activity, respectively. Negative controls (Scramble) were used for both reactions. 12 h following transfection, the transfection medium was replaced with complete medium and was then replaced with AIM when the cells reached 100% confluence to induce adipocyte differentiation. 48 h following adipogenic treatment, the cells were subjected to RNA extraction and qRT-PCR analysis. 72 h following adipogenic treatment, the cells were subjected to protein isolation and Western blotting analysis. For oil-red O staining, the cells were treated with AIM for 72 h, then with insulin alone for additional 48 h.

For the Tgfbr1 loss of function study, we transfected ST2 cells for 12 h with either 30 nmol/L Tgfbr1 siRNAs or negative control siRNA (Genepharma, Shanghai, China) using lipofectamine RNAi-max. For the Tgfbr1 gain of function studies, the ST2 cells were transfected with Tgfbr1 expression plasmid or the empty vector for 12 h using lipofectamine 3000 (Invitrogen, Carlsbad, CA, USA). AIM was added to the cultures to allow the cells to differentiate when reaching 100% confluence.

### Constructs and luciferase reporter assay

The online programs TargetScan (http://www.targetscan.org/), PicTar (http://pictar.mdc-berlin.de/) and miRDB (mirdb.org/miRDB) were used to predict the potential target genes of miR-140-5p. We PCR-amplified the 3′-UTR sequences of the potential target genes carrying the putative miR-140-5p binding sites using mouse DNA as a template. The primers are listed in Supplementary Table 2. The PCR products were subcloned into a modified pGL3-control vector^19^ at EcoRI/EcoRV sites. 293-AD cells were cotransfected with miR-140-5p mimics or its negative control along with the 3′-UTR construct in 24-well plates. The Renilla luciferase reporter plasmid pRL-SV40 (10 ng) was also included in the cotransfection mixture. The cells were harvested, lysed and subjected to luciferase assay 36 h after transfection using a dual-luciferase reporter assay kit (Promega, San Luis Obispo, CA, USA). The relative luciferase activity was calculated by normalizing the Firefly luciferase activity to the Renilla luciferase activity.

For the miR-140-5p promoter study, the wild-type mouse miR-140-5p promoter construct (*miR140/Luc*) was made as described by Yang^25^. The primers are listed in Supplementary Table 2. The DNA fragment obtained by PCR was inserted into pGL3-basic at *Kpn*I and *Hin*dIII sites, respectively. Point mutation to the C/EBP binding sequence (−281nt, CCCTTTCACCAGCC) was made. To study the involvement of C/EBPs in the regulation, the wild-type or mutant miR-140-5p promoter construct was co-transfected with C/EBPα expression plasmid (addgene, Cambridge, MA), or the empty vector into 3T3-L1 cells using lipofectamine 3000 reagent. pRL-SV40 was also included in the co-transfection mixture. 36 h after transfection, the cell extracts were harvested and the luciferase activity was measured.

### Oil-red O staining

Fully differentiated adipocytes were gently washed twice with phosphate-buffered saline (PBS), and then fixed in 4% paraformaldehyde for 10 min. The samples were then washed twice with deionized water, and staining was carried out in 60% saturated oil-red O solution for 5 min. For oil-red O quantification, isopropanol was added to each well. Light absorbance was measured at 520 nm.

### ALP Staining

The ST2 cells were cultured for 14 days followed by alkaline phosphatase (ALP) staining as described in previous studies^37^. In brief, cells were fixed in 10% formalin for 10 min and stained using 1-Step™ NBT/BCIP staining kit (Pierce, Thermo Scientific, Rockford, IL, USA) for 15 min.

### Western blot analysis

Cells were lysed and proteins were separated by SDS-PAGE and transferred onto nitrocellulose membrane. The membranes were incubated overnight with rabbit monoclonal anti-C/EBPα, (Abcam, Cambridge, MA), rabbit polyclonal anti-TGFBR1 (Abgent, San Diego, CA), rabbit polyclonal anti-PPARγ, anti-aP2, or anti-β-actin antibodies (Proteintech, Wuhan, China). This was then followed by incubation with the corresponding horseradish peroxide-labeled IgG (1:3000) for 1 h. Finally, chemiluminescence reagent (Advansta, Menlo Park, California) was used to visualize the results.

### Statistical analysis

Data are expressed as mean ± SD. For the relative mRNA and luciferase activity quantification, the means of the control groups are set to 1. Statistical analysis was performed using the independent t test. A p value of < 0.05 was considered to be statistically significant.

## Additional Information

**How to cite this article**: Zhang, X. *et al.* miR-140-5p regulates adipocyte differentiation by targeting transforming growth factor-β signaling. *Sci. Rep.* **5**, 18118; doi: 10.1038/srep18118 (2015).

## Supplementary Material

Supplementary Information
