# Supplementary material for: miR-140-5p regulates adipocyte differentiation by targeting transforming growth factor-β signaling
Source: Sci Rep. 2015 Dec 11;5:18118. doi: 10.1038/srep18118 (PMC4676041; doi:10.1038/srep18118)
Supplement: Supplementary Information [file srep18118-s1.pdf]

## Supplementary Information

**miR-140-5p regulates adipocyte differentiation by targeting transforming growth factor-β signaling**

Xin Zhang[1§], Ailing Chang[2§], Yongmei Li[1], Yifei Gao[1],

Haixiao Wang[3], Zhongshu Ma[2], Xiaoxia Li[4], Baoli Wang[1]*

[1] Collaborative Innovation Center of Tianjin Metabolic Diseases Hospital, Key Laboratory of Hormones and Development (Ministry of Health), Metabolic Diseases Hospital & Institute of Endocrinology, Tianjin Medical University, Tianjin 300070, China

[2] Division of Endocrinology, Tianjin Medical University General Hospital, Tianjin 300052, China

[3] School of Integrative Medicine, Tianjin Traditional Medical University, Tianjin 300193, China

[4] College of Basic Medical Sciences, Tianjin Medical University, Tianjin 300070, China

[§]These authors contributed equally to the work.

***Correspondence author:** Baoli Wang, MD, PhD. E-mail: blwang@tmu.edu.cn. Tel: 86-22-23542730, Fax: 86-22-23528460

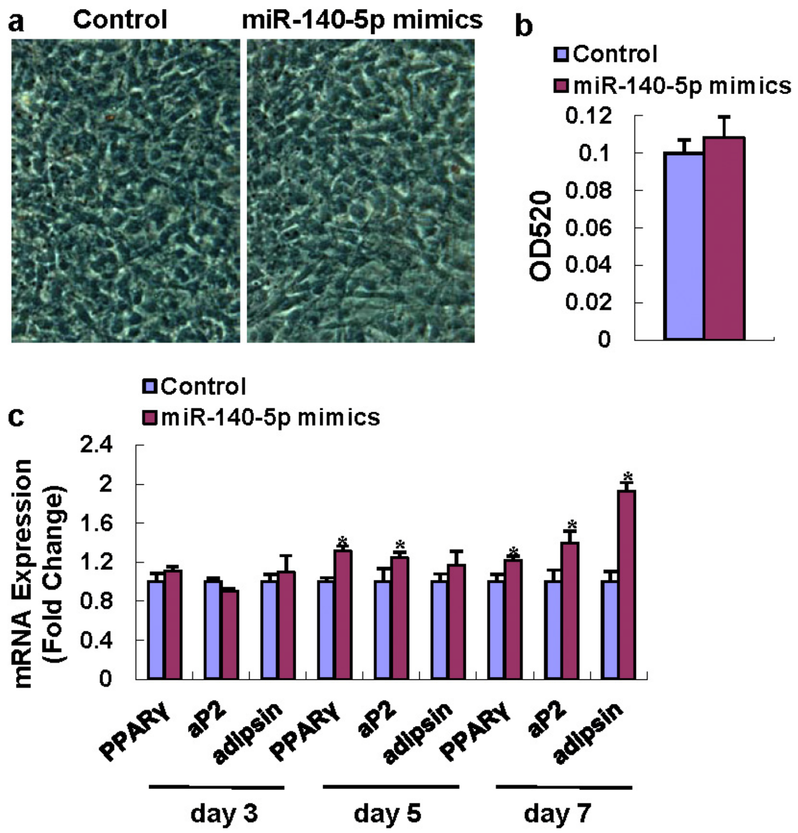


**Figure S1. miR-140-5p mimics did not induce adipocyte formation in absence of adipogenic treatment.** miR-140-5p mimics transfection did not obviously induce adipocyte formation from ST2 in absence of AIM. Oil-Red O extracted with isopropanol was measured at OD520 (a, b). The mRNA levels of PPARγ, aP2 and adipsin were slightly induced at some indicated time points after transfection (c). *Significant *vs.* negative control, $p < 0.05$.

**Table 1 The primers used for RT-PCR quantifications of adipogenic and osteogenic genes**

| Genes | Forward primer sequences | Reverse primer sequences |
|---|---|---|
| PPARγ | 5'-CTTGACAGGAAAGACAACGG-3' | 5'-GCTTCTACGGATCGAAACTG-3' |
| C/EBPα | 5'-CTGATTCTTGCCAAACTGAG-3' | 5'-GAGGAAGCTAAGACCCACTAC-3' |
| aP2 | 5'-AAATCACCGCAGACGACAGG-3' | 5'-GGCTCATGCCCTTTCATAAAC-3' |
| adipsin | 5'-TGATGTGTGCAGAGAGCAAC-3' | 5'-CGTAACCACACCTTCGACTG-3' |
| Runx2 | 5'-TCCTGTAGATCCGAGCACCA-3' | 5'-CTGCTGCTGTTGTTGCTGTT-3' |
| Alp | 5'-CCAGAAAGACACCTTGACTGTGG-3' | 5'-TCTTGTCCGTGTCGCTCACCAT-3' |
| Opn | 5'-GCTTGGCTTATGGACTGAGGTC-3' | 5'-CCTTAGACTCACCGCTCTTCATG-3' |
| Col1a1 | 5'-CCTCAGGGTATTGCTGGACAAC-3' | 5'-CAGAAGGACCTTGTTTGCCAGG-3' |
| β-actin | 5'-AAGACCTCTATGCCAACACAG-3' | 5'-GGAGGAGCAATGATCTTGATC-3' |

**Table 2 The primers used for 3’-UTR and promoter amplifications**

| Genes | Forward primer sequences | Reverse primer sequences |
| --- | --- | --- |
| Tgfbr1 | 5’- AGAATTCTTGCAGCAGTGGAATATGG-3’ | 5’-AGATATCTTCCTTCGGCAATAGACAAG-3’ |
| Wnt11 | 5’-AGAATTCGCTTCAAAGGAAACTGATAG-3’ | 5’-AGATATCACAGATCACATACAGGGAAAC-3 |
| Ostm1(conserve | 5’-AGAGCTCTTAGTCACTGGGAGCCCACT-3’ | 5’-AAAGCTTCTGGCATAAGATGAATCCATC-3’ |
| Ostm1(poorly conserved) | 5’-AGAGCTCAGGAAGGCAGGAGTGTTTGG-3’ | 5’- GAACGCGTGCCTCAGGATTCAGAAC-3’ |
| miR-140-5p promoter | 5’-AGGTACCAGAACAAGGCTAAGCAAAGG-3’ | 5’-CAAGCTTATGAAATCATTCCCACAAGC-3’ |
